# “They cared about us students:” learning from exemplar clinical teaching environments

**DOI:** 10.1186/s12909-019-1551-9

**Published:** 2019-04-29

**Authors:** Althea Gamble Blakey, Kelby Smith-Han, Lynley Anderson, Emma Collins, Elizabeth Berryman, Tim Wilkinson

**Affiliations:** 1 Otago School of Medicine, University of Otago, Dunedin, NZ New Zealand; 2 Department, Bioethics Centre, University of Otago, 71 Frederick St, PO Box 56, Dunedin, NZ 9054 New Zealand; 3 Otago Polytechnic and Staff Nurse, Southern District Health Board, Dunedin, NZ New Zealand; 4 North Shore Hospital, Waitemata District Health Board, Auckland, NZ New Zealand

## Abstract

**Purpose:**

In order to foster positive student experiences in the clinical learning environment, we wanted to better understand which teaching practices they regard highly.

**Methods:**

In 2016, the authors undertook a paper ‘exemplar’ survey (ES) of all fifth year medical students at one tertiary teaching site. Students had experienced all assigned clinical rotations over a two year period. Following a 66% response rate, we identified two clear exemplar clinical areas (ECAs). Over 2016–7, six focus groups with multidisciplinary staff members from these clinical areas were held, with the aim to identify, discuss and understand their specific teaching practices in more detail.

**Results:**

The authors present descriptions of positive student experiences and related staff practices, in five themes. Themes emerged around foundational logistic and personal factors: central to student *and* staff data is that ‘welcome’ on a daily, and ongoing basis, can be foundational to learning. Central to ECA staff data are universal practices by which *all* staff purposefully work to develop a functional staff-student relationship and play a part in organising/teaching students. Students and ECA staff groups both understood teacher values to be central to student learning and that cultivating a student’s values is one of their major educational tasks.

**Conclusions:**

The framework formed by this thematic analysis is useful, clear and transferrable to other clinical teaching contexts. It also aligns with current thinking about best supporting student learning and cultivating student values as part of developing professionalism. Instigating such practices might help to optimise clinical teaching. We also tentatively suggest that such practices might help where resources are scarce, and perhaps also help ameliorate student bullying.

**Electronic supplementary material:**

The online version of this article (10.1186/s12909-019-1551-9) contains supplementary material, which is available to authorized users.

## Background

Get the learning environment right, and the learning will look after itself (Alan Clarke, in [1], p. 85). Medical student experiences in the clinical environment are crucial to their learning; what they are formally taught, see and do shapes their development as a practitioner [2, 3]. Positive experiences might result from a teacher taking time to discuss a clinical case in detail, negative from a teacher asking the student questions which are knowingly too hard for them to answer, with the aim to embarrass or belittle. While the former experience might enhance student learning and development, the latter will almost certainly impede it [4, 5].

Currently, it is understood that a student’s negative experiences in the clinical environment will likely include bullying (mistreatment, harassment, etc. [6–9] as defined by Mavis [6]). Notable cases of bullying feature in recent Australasian media and publications from professional bodies [10, 11]. Together with the current academic literature, these documents indicate that student bullying is a significant, persistent and worldwide phenomenon [7–9]. As a result, the bullied student can suffer negative effects on learning, academic achievement and clinical performance [12], acute and ongoing mental health issues [13] and an overall impeded professional development [14]. These effects can persist over a victim’s entire career [15].

In this article, we describe practices perceived as positive learning experiences by a cohort of medical students and staff from two exemplar clinical teaching areas voted the best by students. We present these practices on their own merits, as such information can offer helpful insights for any clinical teacher in the clinical workforce [16]. We also present these practices because they potentially represent a useful foundational approach to clinical areas suffering resource scarcity, and because of a potential link between these teaching practices and a marked lack of reports about bullying in our exemplar departments. We also report about these teaching practices because of the growing interest in the extent of healthcare management’s duty of care for all employees, now articulated in current NZ workplace health and safety legislation [17–19] which stipulates management’s responsibility to *effectively* tackle workplace bullying.

The exemplar survey (ES, see Additional file 1 for further information) we describe in this article was undertaken prior to, and informed the administration of, a wider action research project called Creating A Positive Learning Environment (CAPLE). This project entailed the development and implementation of an intervention in response to general concerns of medical student bullying in clinical work environments in Australasia.

### Explicating clinical teaching and learning

While an in-depth discussion about the definition of teaching and learning is outside the scope of this article, we still need to explicate what we mean by teaching and learning in the clinical environment (Table 1). This is because sources of learning in clinical practice are diverse, to include many interrelated and contextual practices. We understand that students are taught and learn both explicitly and implicitly from: formal instruction, e.g. a presentation about a clinical case, from observing or participating in activity or process, the use of written materials, and the language, behaviour and actions of clinical staff and patients [3].

**Table 1:** Key points for exemplar teaching practice in the clinical learning environment. On the basis of our findings, we suggest the following

1 Staff in each clinical environment assign time and resources to deliberately make provision for students’ arrival. Provision of a daily deliberate welcome to student into each clinical environment, e.g. by introducing everyone by name in the operating theatre/clinic and briefly explaining the students’ role and current learning aims.	
2 That responsibility for teaching is shared between multidisciplinary and support staff, who understand how to develop and use opportunities for teaching, e.g. teachable moments. Skills in teaching should be a priority focus of staff development.	
3 Teachers create opportunities to develop a healthy relationship with their students, sufficient to enhance their understanding of each students’ specific learning needs.	
4 The potential power of teacher values on student learning (e.g. caring, respect) be understood by staff and be a focus of staff development. Similarly, how a student’s work might be explicitly valued as part of clinical practice.	
5 Teachers understand that cultivating students values, can, and should be done in clinical practice. Specific skills to do this would be a further important focus for staff development.	

## Method

The ES was administered to all 80 fifth year medical students who had completed every training ‘run’ at offered at one tertiary site in Australasia. Student data recorded which departments were the best for their positive learning experiences, and why. Data were also gathered from focus groups held in the two clinical areas voted as the ‘top’ exemplars. The latter data included detailed verbal accounts of teaching practices the exemplar clinical area (ECA) teachers used, and that they considered excellent teaching practices. Data were taken from a total of six focus groups and 15 multidisciplinary staff members.

### Stage 1: exemplar survey

The ES had several goals, two of which are important to the current article:

1. To gain information about which departments in one teaching site offered the best and worst student learning experiences;

2. To allow researchers to hold focus groups with staff of the ECAs, and gain a detailed understanding of their excellent teaching practices.

The ES was a paper-based four-question survey, administered during a whole-class lecture. Questions were:

1. *Identify the clinical areas/teams that you felt offered you the* ***most*** *support for your learning as a medical student.*
2. *Please identify the actions or behaviours that you felt made these areas/teams stand out as* ***supportive*** *for your learning.*
3. *Identify the clinical areas/teams that you felt offered you the* ***least*** *support for your learning as a medical student.*
4. *Please identify the actions or behaviours that you felt made these areas/teams stand out as* ***least supportive*** *for your learning.*

Two researchers (EB) and (AGB) collated data from questions one & three and recorded each under department/or clinical area. The two with the most ‘votes’ became known as our ECAs, and detailed reports from questions two and four informed the thematic analysis we report here.

### Stage 2: exemplar clinical area (ECA) focus groups

Following the collation of student survey results (2016), two researchers (AGB and KSH) undertook three one-hour focus group interviews with staff from the first exemplar clinical area (time and place as convenient to staff). Staff of the ECA who had a significant role in organising/teaching medical students were invited to take part and included nursing, medical and administrative staff. Researchers opened focus group discussions with the broad question: “*Can you tell me why you think your department was voted ‘the best’ for supportive teaching practices in this student survey*?” In 2017, we approached the second exemplar department staff in the same way, and undertook three more focus groups. We did this specifically to seek triangulation between the two ECA departments and strengthen our conclusions.

### Data analysis and representation

We report responses from student ES questions two and four along with data from ECA groups, all of which informed our thematic analysis.

All participant responses were recorded, transcribed and analysed using a general inductive approach [20]. EB and AGB created themes related to medical students’ positive and negative experiences, and ECA staff supportive teaching practices. Each theme was chosen to be representative of a data segment, and each theme was also reviewed and discussed with the wider author group, together with raw data, to evaluate each as a way to clearly explain findings. This process was continued until consensus was reached about each theme and its meaning and at the point that no further categories arose and data were exhausted - the point of data saturation [21]. Methodology was thus emergent and in line with a constructivist epistemology [22, 23]; we sought answers to a research question with little initial idea of what data might reveal.

We present data in themes and summaries to communicate student experiences or ECA staff group consensus and also using quotations, which are verbatim from survey/focus group transcriptions. Some data are edited to preserve participant confidentiality, in which case we to care to preserve meaning by checking edits with the participant concerned. For some data in this study, we have to be exceptionally strict around issues of confidentiality due to concerns that staff and workplaces can be identified easily from what might seem ordinary information.

## Results

### Exemplar survey (ES)

The ES of medical students had a response rate of 66% (54/80 students). Results strongly suggested that students had vastly different experiences in the clinical environments in question. Two clinical areas (ECA 1 & 2) emerged clearly as exemplars, one with 48% of all votes (26/54), out of 22 departments, and a corresponding near-absence (one vote) for this department in terms of unsupportive practices. In contrast, the department voted most unsupportive received 12 votes (22%) with two reports of supportive practices.

One main focus for this paper is ES question two, about supportive teaching practices. This question yielded 155 comments, several of which were general descriptions, without detail to indicate the practice could be easily taken up and used, e.g. “*clear teaching.”* For this article, we report on ES data which was more specific and could more easily guide practice. Our second main focus is data from the six ECA focus groups.

### Student experiences of unsupportive teaching

While data about unsupportive teaching experiences are not the focus of this article, this information can set the scene for the discussion of results about supportive practice. For example, data from survey question four indicate the existence of generally ineffective teaching practices, such as students ‘being told about’ a topic, but being offered little opportunity to apply and consolidate knowledge in a clinical context. A significant proportion of comments (19/91 = 21%) could also be confidently identified as bullying, e.g. verbal abuse: “*I was told I should go back to med school and ask for a refund because I obviously didn’t learn anything…*.”

### Student experiences of supportive teaching

We present teaching practices in five themes, the first two of which emerged from the ES and ECA focus groups; the last three, from ECA staff focus groups.

### Theme 1. ongoing welcome

Welcome practices we describe here were reported by students in the survey and staff in ECA focus groups. Students indicated that they valued acts that helped them feel ‘welcome’ on first arrival in a department and on an ongoing daily basis. Specifically, responses indicated that students felt welcomed because staff seemed to expect them, had learned their names, prepared for their arrival and taken care to ensure each student understood what to do about practical things like storage of their belongings, making hot drinks and where/when to take a break.

*Ongoing* welcome was created by staff carrying out welcoming practices over the students’ entire time in the department, but also by staff asking each student about what they wanted or needed to do to meet specified learning objectives throughout. Similarly, at times that a student took part in a clinical procedure, staff would discuss which part of the procedure the student was interested in or needed to see or practice (31 comments). Some further examples of welcome were given by five different students in the ES:

- *“The doctors actually knew our names.”*
- *“Friendly and welcoming.”*
- *“Actually acknowledging us…Saying good morning.”*
- *“Inviting us to participate.”*
- *“…asking us what we needed to learn.”*
- *“Feeling accepted as part of a clinical team.”*

ECA staff reported understanding that initial and ongoing welcome had a foundational role in student learning, which was why they prioritised it. Here, one staff member makes reference to how welcome might help learning by enhancing student engagement, and another talks about how welcome might impact a student’s overall learning:

*“…if you don’t welcome them, they won’t want to engage, especially because they are at a disadvantage being a learner. Especially at the start where it’s all overwhelming.”*

*“I learn their names just as fast as I possibly can.”*

*“If you don’t welcome them, there is a possibility that their learning just won’t begin and they will miss out. It might be gone forever.”* (2 different staff, ECA 1)

Staff also reported that welcome could be created with seemingly small, simple, but nevertheless important, acts:

- displaying student ID photos before arrival;
- introducing students to all staff on day one;
- ensuring students physically are orientated to the department, including videos of ‘hard to access’ (e.g. high dependency) areas;
- introducing students to staff in each clinical area, daily (e.g. clinic staff) and for each procedure if appropriate (e.g. in operating theatre);
- having a daily focus on ‘which student does what’ to ensure students knew when and where, each procedure (etc.) was to take place, and who was responsible for them.
*“…we ask them about what they are hoping to get out of the run, on their first session, and we try to tie things back to what we have arranged already, and the other students. We let them know that we put together a ‘run’ with their specific expectations in mind.”*
*“…it’s a very clear introductory session that sets the scene for the students learning with us.”*
  (Two different staff, ECA 1)

### Theme 2. collective responsibility

Practices reported under this theme were emphasised universally by all staff from ECA departments. Specifically, staff reported that they all had a role in organising/teaching medical students, to include administration staff. Responsibility for student teaching was therefore collective:

“[admin staff are]…*just great. We couldn’t do any of it without them*.” (Staff, ECA 1)

“…*well I’m just the organiser, but I know it’s important to them to feel a part if the place, and that we are on their side*.” (Administrator, ECA 1)

*“If I’m busy I know that others can, and will, keep the students involved. They don’t just sit around feeling awkward then.”* (Staff ECA 2).

Staff shared out the following tasks:

- organising students’ rosters;
- supervising students doing tasks;
- having students shadow them at the outset of a placement;
- giving tutorials about specific cases, skills and topics;
- helping students understand their role, e.g. what they were allowed to do;
- reassuring students that they could seek assistance from any staff member;
- attending to acute problems (e.g. a student missing out on an important experience).

One staff member summarised what collective responsibility aimed for, and contrasted their aims with experiences of other (non-exemplar) departments:

- **Staff:** *“There should be nothing that they don’t know about, that we haven’t addressed, or that they don’t get to do.”*
- **Researcher:** *“I guess if you didn’t do that, given their position as students, you’d be setting them up to fail, right?”*
- **Staff:** *“It wouldn’t occur to me to not do it, but I see that would happen. In fact I’ve seen it happen other places, and lots.”* (Staff, ECA 1)

### Theme 3. relationship

This theme comprises several related practices which overall seemed to ensure an ongoing functional staff-student relationship, possibly as an extension of welcome. Examples included staff:

- enquiring about, and communicating with each other about a student’s ‘level’ and specific needs/limitations, to ensure appropriate involvement in procedures;
- enquiring about a student’s background, which might indicate possible learning needs (e.g. ethnicity, cultural background, language skills, past learning experiences or qualifications);
- assigning an overall mentor to engage with and discuss progress with a student, deal with outstanding/problematic issues and plan ahead;
- ensuring the mentor remained the same if the student were rostered to the department again. Here, an administrator notes how this relationship is developed and maintained:
  *“We have photo sheets and we keep notes on the person and where they are from and what they are known as rather than lumping them as ‘the students’ to be processed en masse.”* (Administrator, ECA 1)

### Theme 4. teacher values supporting student learning

Approximately half of ES supportive comments from students were about a teacher’s values, and related behaviours:

- “*Being friendly.”*
- *“Consultants being kind on ward round.”*
- *“Great lovely people with empathy.”* (Three different ES students)

Other supportive teacher values reported by students included: being sociable, thoughtful, inclusive, and concerned about student welfare. A specific example of the latter was given by a student who reported being kindly asked about their health and wellbeing following an absence. Teacher values and learning also featured significantly in data from ECA staff:

*“I treat them with respect, and I expect them to treat me with respect.”*

*“It doesn’t preclude me from teasing the heck out of them…they rib me back, and that’s fine, we don’t go over the line… but it makes them feel part of the place and helps them learn.”* (Staff member, ECA 2)

ECA staff reported that they consciously enacted certain values when teaching, in order to help their students learn. In the following quote, a doctor observes a peer *caring* and notes how they saw students perceive this caring, and grow in confidence and learn as a result:

*“The sense that I always get from the consultants involved with teaching is that they really care about the teaching and the students. But when they [students] get it [understand that the consultants care] it’s a ‘tick’ of being accepted and then they tend not to be so shy and then can do more things. It’s vital to their learning sometimes. I know they do it on purpose, because I’ve complimented them* [the peer] *on it before.”* (Doctor, ECA 1)

One specific value that was reported as supportive to student learning was being keen on, or interested in teaching. This theme featured significantly in the survey (12 comments) as a practice supportive for student learning. ECA staff also understood the need for the students to perceive that their teachers are interested in teaching and that this perception would help them learn. Staff also noted a similar effect on learning with ‘valuing’ a student’s work:

*“When their contribution is valued, by the staff and by the patients, this positive feedback makes them grow...”* (Doctor, ECA 1).

Staff talked about efforts they made to get students doing things in the department with a specific aim to show students that their contribution was valued. Staff also wanted students to understand that they:

*“… add value to a department…that we go out of the way to include the student, especially if we feel they are a bit shy. They appreciate it and grow and learn from knowing that...”* (Doctor, ECA 1).

### Theme 5. teaching students’ values for professional practice

This theme was specific to ECA staff, and was about values staff aimed for their students to develop. Specifically, how students might learn values from a teacher’s actions:

*“It’s not* [teaching isn’t only] ***what*** *you know, because you can find that out from a book, but it’s about* ***how*** *you deal with them* [the students] *and your patients and your colleagues.* ***That’s*** *how they learn to be a good physician* [their emphasis]”

Teachers also explained why they would necessarily address persistent behavioural issues they understood to be related to students’ values:

*“…if they* [the student] *are repeating the same* [bad/inappropriate] *behaviour over and over…we bring it up, and talk about it, help them….by the end of the roster they will be a lot better…It’s about educating the whole person as a doctor…they might end up treating us, or our children!’… ‘Its future- proofing in a way…they are going to run into problems later in their career if we don’t…we* ***have*** *to teach them values* [their emphasis]*.”*

ECA also staff talked about how they needed to teach positive, requisite values implicitly, by modelling ‘being a good person.’ A specific example of this was given by a doctor who had witnessed a student’s distress when ‘pimped’^1^ by another staff member. This staff member, according to this doctor, was “*not showing the values that we would like a student to learn*.” In response, the doctor took time to ask the student how they were and to explain how the other staff member had acted poorly. This doctor indicated that his purpose in doing so was to “*make sure that they are OK”* but also specifically to indicate that “*caring is what we do here*. *This is what they* [students] *need to learn.”* In other words, this doctor deliberately acted in a way that aimed to cultivate the student’s values desirable for clinical practice, instead of the undesirable that are likely from such an incident.

## Discussion

We wanted to understand what exemplar clinical departments did for student teaching and learning in one teaching hospital. The two departments voted exemplars offered a significantly more positive experience for students than most others, complemented by a deficit of reports of student bullying incidents.

We supplement our discussion of each theme we identified in data with examples of what might happen to student learning if the practice was not employed. Some examples form part of the unsupportive ES data, others are summarised from discussions with focus group participants or between our researchers. Our themes for exemplar student teaching are:

1. Ongoing welcome
2. Relationship
3. Collective responsibility
4. Teacher values supporting student learning
5. Teaching students values for professional practice

One important overall observation about our findings was their surprising simplicity. Exemplary teaching was created and supported by a combination of fundamentally logistic and personal factors; specific pedagogic method or theoretical standpoints were not reported as important to student learning. This result is consistent with literature [24, 25] in which logistic and personal practices are confirmed as foundational to much of student learning in the clinical environment, and that the attitudes of clinical teachers can have a positive influence on personal development [14]. We thus find a confirmation that practices reported here should be our main aims in the clinical environment and perhaps a reassuring focus where resources are in demand.

### Ongoing welcome

Our first theme, about ongoing welcome, is well supported by the literature, in which reports indicate that practices related to student ‘welcome’ are essential for some student learning. For example, that welcoming a student at the outset of, and throughout a student’s clinical placement, can be likened to their introduction to and progressive legitimate peripheral participation (LPP) in the workplace, as described by Lave and Wenger [26]. This well-accepted theory enables us to explain how a learner can be effectively and progressively inculcated into workplace practice, in parallel with learning, to progress from newcomer to experienced. Overall, this gradual exposure to the tasks, vocabulary and organisation of a workplace community can support and integrate the student and their learning in a workplace within a specific, perhaps complex, context.

Lave and Wenger’s [26] theory of LPP also helps explain how a learner who becomes separated from a learning community can experience substantially limited learning and professional growth. This view is confirmed by others (e.g. Sheehan, et al., [24]) who describe how a lack of welcome can decrease learner engagement to an extent that some students question their current career choice. Whilst damaging to learning in itself, a student who questions their career choice can also have an impact on patient care, e.g. by unwittingly displaying their unhappiness in body language which is negative or inappropriate. We also understand that neglecting to welcome a learner might also preclude them from:

- understanding the expectations of their new role;
- opportunities for learning at all levels
- clinical discussions and associated learning;
- practicing knowledge application;
- developing skills to a competent level;
- growing confidence in clinical and interpersonal skills.
- feelings of belonging and associated positive emotions [3].

### Relationship

Again, the theme about relationship is not about a specific teaching method, rather a fundamental and foundational basis for all teaching practice, and once more supported by the literature. For example, that such a relationship supports learning by allowing a teacher to understand student-specific learning needs and issues which require attention (e.g. fear of talking to patients) [27–31]. It is also suggested that relationship can be a principal mediating factor in how a student learns from the hidden curriculum [32], and as such, relationship could also be important to learning the fundamental premises of best practice in a personal sense. For example, relationship can mediate a student’s learning about interacting with others as an essential skill for good practice:

The relationship between teachers and learners can be viewed as a set of filters, interpretive screens, or expectations that determine the effectiveness of interaction between teacher and student . . .within [effective] relationships, learners are willing to disclose their lack of understanding rather than hide it from their teachers; learners are more attentive, ask more questions, are more actively engaged . . . learning is contextual, and one of the most important contexts for human beings is other people… [33].

Similarly, Telio et al., [34] discuss teacher-student relationship in terms of creating an educational alliance with a student, and how the resultant relationship can better enable honest, helpful feedback, which a learner takes seriously and puts into action. Importantly, it has also been shown that a student in such a relationship can go on to develop a sense that their teacher is credible and therefore of value to their learning. Again, we see the development of relationship between student and teacher offering several benefits to learning, but also to a student’s personal development.

When we examine teacher-student relationships (however defined) that are ‘wrong,’ ‘negative’ or even non-existent, we gain further insight into how relationship is important to learning and personal development, but also to the work environment in a general sense. Specifically, that failure to establish a positive teacher-student relationship may preclude a student from:

- having their specific learning needs understood and met;
- receiving accurate feedback in a manner that is helpful to learning;
- learning effective communication skills by interacting with the teacher;
- experiencing a sense of belonging in the workplace and its related positive emotions;
- communicating an acute worry to their teacher, say, about a patient’s condition, which can lead to a student becoming implicated in inefficiency, error, or negative clinical outcomes [3].

### Collective responsibility

Collective responsibility for student teaching again included practices which supported student learning in a practical sense, e.g. by enhancing access to learning opportunities, teaching staff, chances to learn from a multidisciplinary team [35] and for ‘teachable moments’ to be used as a way to optimise teaching in a time-poor or unpredictable environment [35]. Pratt, et al. [36] specifically recommend such an approach to teaching; that a focus on teaching and learning should extend beyond the singular use of one attending physician or preceptor, and argue that student learning should be a product of the broader context of learning: the “cultural arena within which engagement is invited and supported, or denied.” (p. 136, see also [37]). Interestingly, data we describe under this theme had a comparative lack of student data. We ascribe this lack to the relatively invisible or background involvement of much of the work done by staff (‘behind the scenes’). As such, we understand that an appreciation of collective responsibility for teaching may well be expressed by students as part of comments about the generally supportive environment and provision of learning opportunities. If responsibility for student learning is not undertaken as a collective, we understand that:

1. student teaching workload would be shared between fewer staff, who might therefore experience relatively more teaching-related stress;
2. students would likely have limited contact with teachers’various teaching methods and knowledge, and experience more limited overall learning;
3. teaching and its administration (e.g. filling out feedback forms) might need to be neglected to meet patient demands;
4. teaching might not happen if a staff member is on leave, busy with their own study or in an acute patient situation;
5. staff with appropriate expertise might not have a chance to divest this to student, e.g. a midwife will have expertise appropriate for medical student learning [3].

### Teacher values supporting student learning

Teacher values in learning is also a topic which has become established in the literature, and one which is enjoying somewhat of a resurgence [44]. ECA staff seemed aware that their values could be perceived by their students, and that some values can help student learning. This view is strongly supported by Palmer [39] who specifies that values such as ‘caring’ can help students’ learning by ameliorating anxiety or fear, and thus enhancing engagement [30, 31, 39–41]. Tanner [41] explains the relative importance of teacher values in learning, and that:

“…how little great teaching has to do with technique and how much it has to do with the teacher as a person.”

ECA data also make specific reference to ‘valuing teaching’ as being supportive for student learning. While such a finding seems self-evident - that someone who values teaching would likely enjoy it, and this would probably positively affect learning - the appearance of valuing teaching in this study was notable, and is a phenomenon rarely explicitly mentioned in other literature. This finding thus offers us an additional understanding of how particular teachers can help students learn better as well as adding weight to more general reports [42] about values supporting teaching. That is, a focus on ‘teaching the teachers’ about teaching & learning and therefore encouraging their ‘valuing of it’ would be an excellent focus point for staff development.

ECA staff also reported valuing students’ work as important to student learning. This value is again seldom seen reported in literature. In the case of this study, we found it tempting to dismiss this value as a practice that simply helped overcome effects of resource constraints. After all, teachers described how students would make a specific practical contribution to the running of the clinical workplace, e.g. by teaching students to be very confident with cannulation and getting them to do ten cannulations on surgery day, to help with student learning but also helping to keep surgeries on schedule. However, we detected something more to the ‘valuing’ of student learning than a practical solution; staff indicated that this ‘positive’ perception of the student offered the student confidence in the workplace, perhaps to progress in ways suggested by Lave and Wenger’s theoretical periphery.

Again, examining what might happen if a student’s work is not ‘valued’ offers insight. Failing to value students work might preclude them from:

- Feeling part of a workplace and experiencing the positive emotions as a result of this;
- Feeling that they have progressed or can contribute meaningfully to patient care;
- Being allowed to undertake new procedures or tasks, or practicing to a competent level [3].

### Teaching students values for professional practice

Cultivating students’ values is another well-established topic in the literature which has now begun to reveal some ways to foster an effective discourse for doing so [30, 38, 39]. This literature supports our staff participants’ understanding that cultivating students’ values can [42] and should [43] be developed as part of their clinical education, and as an important part of developing students’ professionalism [14, 44]. Our ECA teachers also reported that values can be challenging to teach in an explicit sense, but also that values could be taught by acting according to these values, viz., by more than modelling [45], but simply ‘being.’ Some call this teaching by ‘default’ [46, 47], and also note that this approach to teaching and learning is likely to be a pervasive practice:

Observing role models to help us imagine, define, and practice the kinds of behaviours we would like to exhibit in our own lives is one of the most common means by which we learn [46].

Evidence of teaching and learning by default was also, sadly, found in comments we gathered from the question about ‘unsupportive teaching practices’ in our ES. To summarise some of these, and our own thinking, failure to teach values, even if by simply not modelling or ‘being’ a positive value, might mean a student is precluded from:

- developing some values necessary for best practice;
- learning to express these values and behave in ways acceptable to quality practice;
- developing values which are (perhaps unwittingly) modelled to them, such as those unconducive to good practice, e.g. impatience.

### Strengths and weaknesses of our study

Data in the current study contain reports of teaching practices by proxy, i.e. reported through students and not observed first hand. Such reports might be susceptible to a degree of personal interpretation; our understanding of actual teaching practices would be enhanced by direct observation. However, our aim was to determine students’ experiences, and thus we suggest this potential limitation has little impact on our conclusions. Reassuringly, we find confidence in our interpretation as meaningful, as data triangulate well between students and staff, and the two clinical areas approached as part of this study.

We also acknowledge that we make an assumption about supportive experiences of teaching: that ‘supportive’ means helpful for student learning. For example, a student might perceive a practice by which a teacher gives them all the answers as supportive. However, such a practice might be one which does not help the student to reason answers for themselves (see Delany & Golding [48]), which might be what they need to take their learning further. Survey data do not allow us to further tease out distinguish such detail from these reports.

### Further research

Of note in the ES was the near-absence of survey reports about bullying in the ECAs. This absence may be attributable to factors such as an absence of staff with predisposing personal characteristics [4]. Further investigation is required to determine whether the instigation of specific teaching practices may also be responsible for the apparent absence of bullying behaviours. We suggest this be done by measuring change in bullying incidence in a department that adopts the framework of clinical teaching methods reported here. We might also consider including an approach specifically aimed to enhance teaching and learning of values, within such a framework [38].

A more detailed understanding of how valuing a student’s work might translate into better learning in practice is also required. Such an understanding offers an opportunity to further hone a more detailed strategy for foundational learning in the clinical workplace.

## Conclusion

Reports of teaching practices from our ES indicate that, in two specific departments, students experienced teaching practices that were considerably more supportive to student learning than in some other departments in the same hospital. Together, data from the ES and the ECA focus group indicate foundational practices to support the cultivation of skills of practice, knowledge and the values required for excellent clinical practice. Specifically, we find that valuing the work our students do in the clinical workplace, and valuing teaching, might further positively influence learning. Teaching practices reported here are supported by the current literature, to also include very recent literature about teaching students values as a specific, important aim.

We see staff respond to the challenges of practice and teaching with a unified, feasible, pragmatic and effective strategic plan for optimising clinical teaching in a busy and unpredictable environment. While some of the more ‘human’ elements of our findings might be harder to manipulate, as Clarke (see [1]) indicates, it might be that if we look after the learning environment, learning might indeed more easily look after itself.

## Additional file

Additional file 1: Exemplar Questions. (DOCX 839 kb)

## Abbreviations

AGB: Althea Gamble Blakey (researcher and primary author)
EB: Elizabeth Berryman (researcher and co-author)
ECA 1 & ECA 2: 1 and 2 are used to differentiate between the two clinical departments (correspondingly approached in 2016 & 2017) used for our focus groups about exemplar clinical areas
ECA: Exemplar Clinical Area – a clinical department or ward voted in the ES to be comparatively better for student learning experiences
ES: Exemplar survey: A survey of medical students undertaken to understand which department offered the most and least student learning experiences in one teaching hospital
KSH: Kelby Smith-Han (researcher and co-author)

## Footnotes

1 Being deliberately humiliated by a teacher asking questions that are too hard [5].
