# Supplementary material for: “They cared about us students:” learning from exemplar clinical teaching environments
Source: BMC Med Educ. 2019 Apr 29;19:119. doi: 10.1186/s12909-019-1551-9 (PMC6489296; doi:10.1186/s12909-019-1551-9)
Supplement: Supplementary file 1 — Exemplar Questions. (DOCX 839 kb) [file 12909_2019_1551_MOESM1_ESM.docx]

**Exemplar Survey questions (students)**

**Confidential**

1. Identify the **clinical areas/teams** that you felt offered you the **most support** for your learning as a medical/nursing student?

2. Please identify the most important actions or behaviours that you felt made these areas/teams stand out as supportive for your learning?

*Please turn over*

3. Identify the **clinical areas/teams** that you felt offered you the **least support** for your learning as a medical/nursing student?

4. Please identify the actions or behaviour that you felt made these areas stand out as being least supportive for your learning?

**Focus group for clinical staff from the supportive area/team:**

Your clinical area has been identified by medical students as being the most supportive of student learning.

1. Why do you think your area or team was identified as such?

2. Can you tell us about the structure of your area/team?

3. Can you describe how staff in your area/team communicate?

4. How do problems get resolved within your team/area?

5. What actions do staff take in your area or team to make students feel welcome?

6. What do you think your clinical area/team does well when it comes to creating an effective student learning environment?

7. What possible barriers might prevent your clinical area/team from achieving an effective learning environment?
